# The role of magnetic resonance imaging in the evaluation of bone tumours and tumour-like lesions

**DOI:** 10.1007/s13244-014-0339-z

**Published:** 2014-07-09

**Authors:** Duarte Nascimento, Guilherme Suchard, Maruan Hatem, Armando de Abreu

**Affiliations:** 1 Serviço de Imagiologia do Serviço de Saúde da Região Autónoma da Madeira, Avenida Luís de Camões, nº 57, 9004-514 Funchal, Portugal; 2 Serviço de Radiologia do Hospital Mãe de Deus, Rua José de Alencar, 286, Menino Deus, Porto Alegre, RS, 90880-480 Brazil

**Keywords:** Magnetic resonance imaging, Bone neoplasms, Diagnosis, Neoplasm staging, Follow-up

## Abstract

Bone tumours and tumour-like lesions are frequently encountered by radiologists. Although radiographs are the primary screening technique, magnetic resonance imaging (MRI) can help narrow the differential or make a specific diagnosis when a lesion is indeterminate or shows signs of aggressiveness. MRI can extend the diagnostic evaluation by demonstrating several tissue components. Even when a specific diagnosis cannot be made, the differential diagnosis can be narrowed. MRI is superior to the other imaging modalities in detecting bone marrow lesions and tumoral tissue (faint lytic/sclerotic bone lesions can be difficult to visualise using only radiographs). Contrast-enhanced MRI can reveal the most vascularised parts of the tumour and MRI guidance makes it possible to avoid biopsing necrotic areas. MRI is very helpful in local staging and surgical planning by assessing the degree of intramedullary extension and invasion of the adjacent physeal plates, joints, muscle compartments and neurovascular bundles. It can be used in assessing response to neoadjuvant therapy and further restaging. The post-therapeutic follow-up should also be done using MRI. Despite the high quality of MRI, there are a few pitfalls and limitations of which one should be aware. Applications of MRI in bone tumours will probably continue to grow as new sequences are further studied.

• *When a lesion is indeterminate or shows signs of aggressiveness*, *MRI is indicated*.

• *When MRI does not lead to a diagnosis, biopsy is indicated*.

• *MRI is superior to the other imaging modalities in detecting bone marrow lesions*.

• *MRI is very helpful in local staging and surgical planning*.

• *MRI is used in assessing the response to neoadjuvant therapy*, *restaging and post*-*therapeutic follow*-*up*.

## Introduction

Radiographs are the primary screening technique used for bone tumours and tumour-like lesions [1]. When a lesion is indeterminate or shows signs of aggressiveness, magnetic resonance imaging (MRI) is indicated for further characterisation [1]. It can extend the diagnostic evaluation by demonstrating components such as cartilage, vascular tissue, fat, liquid and haemosiderin. Even when a specific diagnosis cannot be made, MRI can help by narrowing the differential diagnosis. These are the reasons why MRI has changed from a single study-based diagnosis (solely based on radiographs) to a multimodal imaging approach (which now includes MRI).

Faint lytic/sclerotic bone lesions can be difficult to visualise using only radiographs. MRI is superior to the other imaging modalities in detecting bone marrow lesions [2].

Aggressive indeterminate cases will require histological confirmation before proceeding to staging and establishing a therapeutic approach. The high percentage of biopsy tract contamination [3] indicates that this track should be included in the surgically removed area. Contrast-enhanced MRI (CEMRI) can reveal the most vascularised parts of the tumour and MRI guidance makes it possible to avoid biopsing necrotic areas [2].

MRI is very helpful in local staging and surgical planning because it assesses the degree of intramedullary extension (and dimensions) and invasion of the adjacent physeal plates, joints, muscle compartments and neurovascular bundles.

Restaging after neoadjuvant therapy and the post-therapeutic follow-up should also be done using MRI.

Our purposes are: (1) to discuss MRI features that can help narrow the differential or make a specific diagnosis of bone tumours and tumour-like lesions; (2) explain why MRI is the optimal imaging method for sensitive detection of tumoral tissue, local staging, preoperative evaluation, assessing the response to neoadjuvant therapy, restaging and follow-up, and (3) to discuss potential pitfalls and limitations.

## MR imaging sequences and protocol optimisation

In musculoskeletal (MSK) MRI, the T1 signal intensity is described by comparison to that of muscle. Although many argue that muscle should also be the standard reference for T2-weighted imaging, using fat as the reference can be helpful, particularly in anatomic regions where there is relatively little muscle (e.g. fingers and toes).

### T1-weighted imaging (T1WI)

T1WI is very important in the evaluation of bone marrow. Most bone tumours will be evident as lesions with low signal against a background of surrounding fatty marrow [4].

T1WI also provides excellent contrast among the cortical, marrow and surrounding tissues [4].

### Fat suppression, T2WI and STIR

The use of fat suppression (FS) in MRI can confirm or exclude the presence of fat in a lesion (this is particularly useful for diagnosing haemangioma and lipoma).

Water shows higher signal than fat on T2WI, but suppressing the fat signal can allow an even better evaluation of the extent of oedema.

Suppressing the fat signal in T1WI after injection of gadolinium-based contrast medium increases the conspicuousness when assessing tumour vascularisation.

Short tau inversion recovery (STIR) sequences effectively and homogeneously suppress all fat signal but cause tissues with T1 signal similar to or greater than that of water to show hyperintensity. This can lead to overestimation of the tumoral extension and compromise its characterisation [4].

### Chemical shift imaging

Chemical shift-based fat suppression can help evaluate bone marrow infiltration [5]: it increases the sensitivity for detection of metastases and myeloma lesions and may be used to improve specificity when equivocal marrow changes are seen on MRI [6]. Tumoral tissue will not show signal loss in opposed-phase images, in contrast to normal fatty or haematopoietic red marrow.

### Using intravenous gadolinium-based contrast medium

**Fig. 1:**
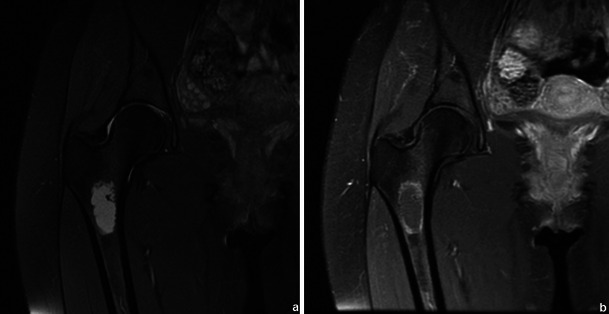
This femoral lesion showing high signal in T2FS WI (**a**) could be solid (fibrous dysplasia) or liquid (solitary bone cyst). T1FS gadolinium-based contrast medium-enhanced sequence (**b**) showed peripheral enhancement, typical of liquid content, favouring the diagnosis of a solitary bone cyst

Most bone tumours and tumour-like lesions have a significant amount of cartilaginous tissue (hyperintense on T2WI). CEMRI can be used in the differentiation between solid hyperintense and fluid-containing lesions. Solid, non-necrotic areas will show diffuse enhancement while liquid will not (Fig. 1).

**Fig. 2:**
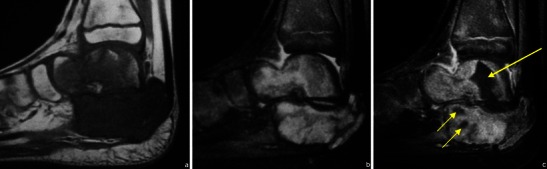
T1WI (**a**), T2FS WI (**b**) and CEMRI (**c**) images of a Ewing’s sarcoma involving the talus and calcaneus. Non-enhancing components (arrows in **c**) are related to areas of necrosis

Gadolinium-based contrast medium also helps distinguish oedema from viable tumour (Fig. 2) and allows an accurate determination of the degree of vascularisation.

Fast acquisition techniques after gadolinium-based contrast medium injection allow contrast-enhanced dynamic imaging. Time to peak, maximum enhancement, slope (degree of enhancement during the first pass), washout rate and the shape of the signal enhancement-time curve can be determined. Contrast-enhanced dynamic imaging is said to be approximately 80 % accurate in differentiating benign tumours from malignant ones [7].

The basic principle that viable active tumoral tissue shows a higher degree of enhancement than nontumoral/necrotic areas can also be applied to directing biopsies to viable cellular areas and noninvasive assessment of response to therapies such as radiotherapy and chemotherapy. The most reliable indication of necrosis in MRI is probably still simple: a visible lack of enhancement after the administration of gadolinium-based contrast medium.

### Diffusion-weighted imaging (DWI)

**Fig. 3:**
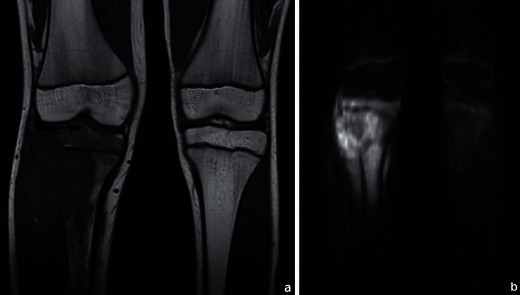
Osteosarcoma of the tibia: coronal T1WI (**a**) shows a large hypointense lesion in the proximal tibia, which breaks through the bony cortex and invades the adjacent soft tissue. Corresponding DWI (b = 700 s/mm^2^) (**b**) reveals restricted proton diffusion, consistent with malignancy. Tumoral necrosis (in the centre of the tumour) displays a less hyperintense signal. (Images published by Markus U., Reichardt W. and Kontny U. in MRI: New Developments in Bone Tumor Imaging. Magnetom Flash 2/2011© used with permission of Prof. U. Markus)

DWI shows restriction to the diffusion of water molecules in malignant tumours [4] (Fig. 3). A favourable therapeutic response is associated with a decrease in the signal intensity in high b values [8]. Apparent diffusion coefficient (ADC) ratios may also be used for assessing the response to neoadjuvant therapy [9]. The use of DWI however is still not included in the guidelines for routine evaluation of malignant tumours [10].

### MR spectroscopy imaging

Wang et al. [11] showed elevation of the choline peak in the majority (18 of 19 patients) of malignant bone tumours and also concluded that spectroscopy can help differentiate malignant from benign tumours by revealing the presence or absence of water-soluble choline metabolites. Further investigation is needed [1], but MR spectroscopy seems to show great potential in differentiating benign from malignant lesions.

### Establishing appropriate protocols

Every institution should have established protocols for the MSK imaging evaluation of different anatomic locations (which should include proper imaging planes for the specific region studied).

An important issue relates to the fact that most institutions do not routinely use contrast in general MSK studies (a significant number of lesions are detected incidentally). Some even consider that the routine use of gadolinium in the initial MRI evaluation of a possible primary MSK neoplasm is not justified [12]. We however recommend using CEMRI whenever the radiographs and conventional (T1 and T2FS) MR sequences do not provide an obvious specific benign diagnosis. Even when a specific diagnosis is reached, all lesions raising suspicion for malignancy should still be evaluated after gadolinium-based contrast medium injection in order to optimise the border characterisation and separate oedema from active tumour.

MRI findings should always be correlated with a radiographic study. If there are none available, they should be obtained before giving a final report.

## Applying MRI in tumour detection and diagnosis

(1) Accurate lesion detection

**Fig. 4:**
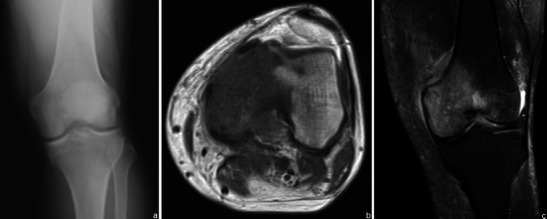
Patient with chronic left knee pain. Radiographs were obtained (**a** normal frontal radiograph). Subsequent MRI study showed abnormal signal intensity in the bone: **a** low T1WI signal (**b**) and T2FS WI hyperintense lesion (**c**) related to a bone lymphoma

**Fig. 5:**
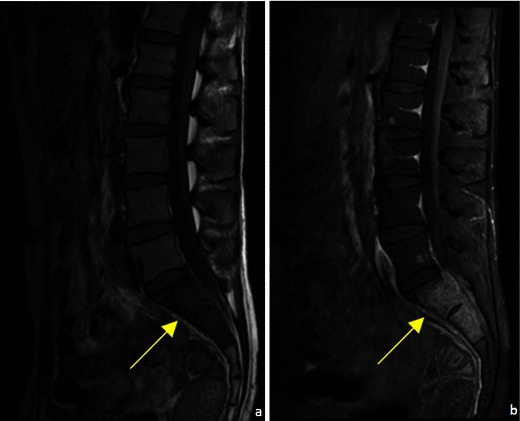
A 28-year-old patient with a testicular neoplasm and spinal metastases. T1FS WI showed abnormal signal in the superior sacral elements (arrow in **a**). CEMRI showed diffuse enhancement of the superior sacral elements (arrow in **b**) with spinal cord extension and focal lesions in the lumbar vertebrae

Faint lytic/sclerotic bone lesions can be difficult to identify using only radiographs. MRI is superior to the other imaging modalities in detecting primary and secondary bone lesions [2] because it can conspicuously show areas of abnormal signal intensity within a bone that should contain normal fatty marrow [13] (Figs. 4 and 5).

In suspected cases of pathological fractures, MRI should be used to evaluate the existence of an underlying lesion [1].

(2) MRI evaluation: aspects favouring benignancy or malignancy

**Fig. 6:**
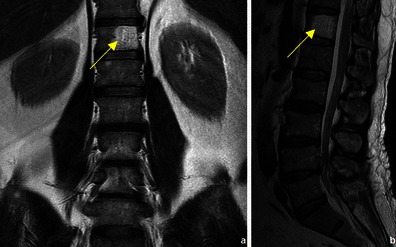
Coronal T1WI (**a**) and sagittal T2WI (**b**) show a well-circumscribed vertebral lesion, with signal intensity similar to that of the subcutaneous fat: vertebral haemangioma

**Fig. 7:**
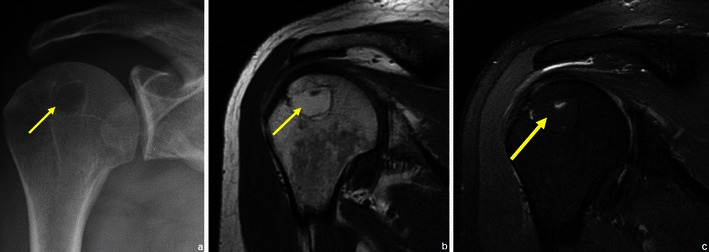
Patient with a humeral lipoma (arrows in **a**, **b** and **c**): frontal radiograph shows a lytic lesion in the head of the humerus. This lesion showed signal intensity similar to that of fat on T1WI (**b**). The signal was suppressed in T2FS WI (**c**)

Both benign and malignant tumours usually have inferior signal intensity to the normal marrow signal in T1WI [13]. This is why MRI does not allow accurate prediction of the malignancy or benignancy of a lesion based solely on its signal intensity [13]. There are however some features that, if present, can be highly predictive of a benign etiology, such as the presence of normal fatty marrow [14] or fat within a bone lesion (a common feature in vertebral haemangioma (Fig. 6) and in lipoma (Fig. 7).

MRI can also confirm the presence of fatty or haematopoietic marrow in areas of normally sparse trabeculae (e.g. greater tuberosity of the proximal humerus), which can simulate lytic lesions on radiographs or CT [15].

Typically, benign lesions are well defined and sharply demarcated from the surrounding healthy tissue. Malignant lesions are usually extensive and involve surrounding tissue to a greater extent.

**Fig. 8:**
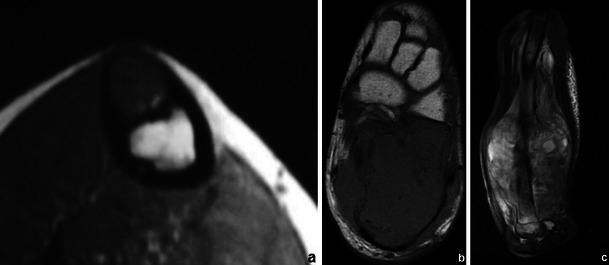
Associated soft tissue component in two different cases of Ewing’s sarcoma: one of the calcaneus (**a**) with medial cortical destruction and one of the tibia (**b**) with anterior cortical disruption

Benign fibrous or expansive lesions can give the false impression of cortical destruction in radiographs (e.g. aneurysmal bone cyst, giant cell tumour and chondroblastoma). The opposite can occur with malignant lesions. MRI can help exclude true cortical destruction (Fig. 8).

The presence of an associated soft tissue component is one of the most important imaging malignancy predictors in bone tumours. It can point to a malignant degeneration of enchondroma, osteochondroma, previous irradiated areas and Paget’s disease. This is usually a prominent feature in Ewing’s sarcoma (Fig. 8) and osteosarcoma.

Although osteomyelitis can have an aggressive permeative aspect in radiographs, it usually does not have an associated solid soft-tissue mass [16]: MRI can help distinguish it from true permeative neoplasms such as Ewing’s sarcoma.

The absolute value of tumour-associated oedema in the diagnosis is limited, as its degree often does not correlate with the degree of malignancy or tumour aggressiveness [17].

**Fig. 9:**
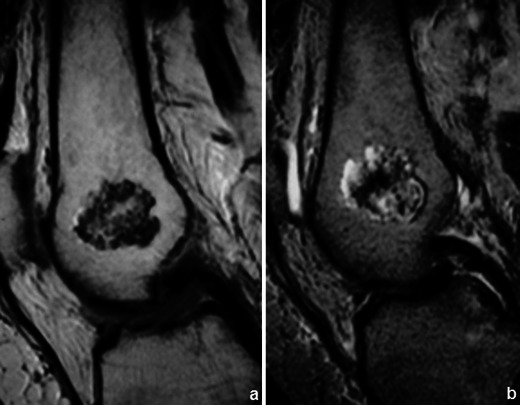
Typical enchondroma: small lesion located in the distal femur with low/intermediate signal on T1WI and hyperintense on T2WI, containing calcifications, with no associated soft tissue component or adjacent oedema

**Fig. 10:**
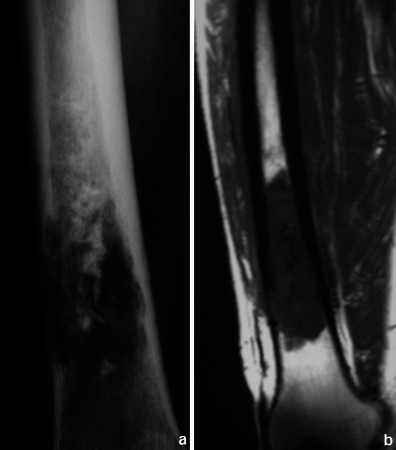
This patient had chronic lower thigh pain. The radiograph (**a**) showed an area of bone destruction with central calcification. Coronal T1WI clearly showed a hypointense lesion extending beyond the disrupted cortical tissue. This lesion was a chondrosarcoma

In some cases, differentiating an enchondroma (Fig. 9) from a low-grade chondrosarcoma (Fig. 10) can be a difficult task for both radiologists and pathologists. Many important suspicious imaging features can be assessed using MRI (Table 1).

**Table 1:** List of clinical and imaging features used in the differential diagnosis between enchondroma and chondrosarcoma

Enchondroma	Chondrosarcoma
More common in extremities	More common in the axial skeleton
No pain (only if associated with fracture)	Can cause pain
No periostitis	Can have associated periostitis
No growth beyond skeletal maturity	Rapid growth, regardless of the skeletal maturity
No soft tissue component	Associated focal cortical thickening
Absence of bone oedema	Different patterns of signal intensity
Size usually ≤4 cm	Bone destruction
	Loss of calcifications on follow-up
	Soft tissue component
	Bone marrow oedema
	Size usually >4 cm

MRI can also make a difference when evaluating lesions located in the bone surface by determining whether they penetrate the cortical and invade the medullary region [16].

**Fig. 11:**
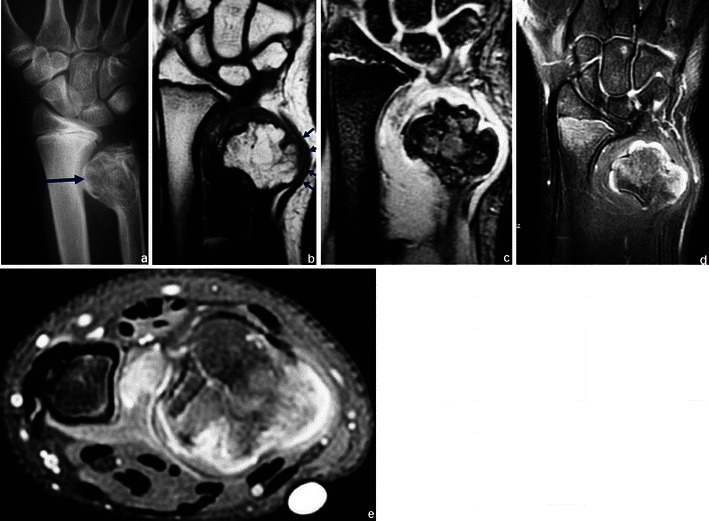
Anteroposterior radiograph (**a**) and corresponding MRI study (coronal T1 (**b**), coronal T2FS (**c**) and coronal (**d**) and axial post-Gd T1WI (**e**) of a broad-base osteochondroma of the ulna. T1WI shows normal bone marrow signal intensity inside the lesion, and T2FSWI shows adjacent effusion and oedema of the adjacent soft tissues. The flexor carpi ulnaris tendon is compressed and displaced (arrows in **b**). The axial post-Gd T1WI (**e**) shows no compression of the adjacent neurovascular structures

**Fig. 12:**
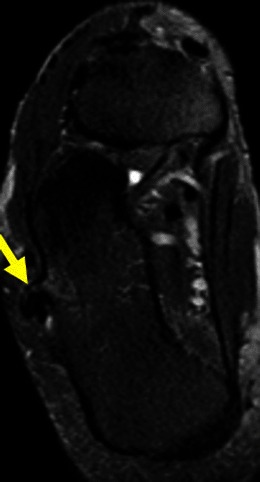
Young female with ankle pain. This axial T2FSWI shows a small pedunculate osteochondroma of the right calcaneus (arrow) compressing the adjacent fibular tendons

**Fig. 13:**
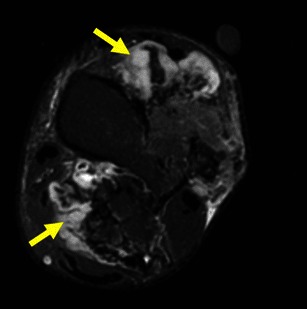
Axial T2FS WI shows a voluminous lesion with a thick cartilagineous cap: a secondary chondrosarcoma of the left distal tibia

Osteochondromas show an uninterrupted medullary and cortical continuity with the parent bone (Fig. 11). Radiographs often do not allow an accurate evaluation of these features, which is why they should be assessed using CT or MRI. Their presence is helpful in excluding other less common tumours and tumour-like conditions (e.g. osteoma, periosteal chondroma, juxtacortical and soft tissue osteosarcomas and myositis ossificans) [16]. A cartilage cap is often demonstrated, which has low to intermediate SI on T1WI and is hyperintense on T2WI. Several complications can occur in osteochondroma, including osseous deformity, fractures (of the lesion or of adjacent bones), compression of adjacent structures (tendons [Figs. 11 and 12], vessels and nerves), bursitis and rarely [18] malignant degeneration (Table 2; Fig. 13). MRI represents the most valuable imaging modality in symptomatic cases because it can demonstrate typical features of associated soft tissue pathology [18, 19]. The cap thickness is the best predictor of malignant change [17]. The normal upper limit for the cartilagineous cap thickness in MRI is usually considered to be 1 cm [16] although younger patients in active growth can reach a width of 3 cm [19].

**Table 2:** List of clinical and imaging features used in the differential diagnosis between osteochondroma and secondary chondrosarcoma

Benign osteochondroma	Secondary chondrosarcoma
No pain (only for fracture, bursitis or compression of adjacent structures)	Can cause pain
No growth beyond skeletal maturity	Rapid growth, suspicious especially if after skeletal maturity
	Presence of calcifications (signal voids) beyond the stalk
No associated soft tissue mass	Presence of an associated soft tissue mass
Thin cartilagineous cap (≤1 cm)*	Thick cartilagineous cap (>1 cm [16])

Flow voids have been demonstrated in vascular tumours (e.g. haemangioma and haemangioendothelioma), renal cell and hepatocellular carcinoma metastases and are uncommon in other tumours [17]. They can therefore be regarded as an additional diagnostic feature but do not have a prominente role in the differentiation of benignancy vs malignancy.

(3) MRI evaluation of fluid-fluid levels (FFLs)

**Fig. 14:**
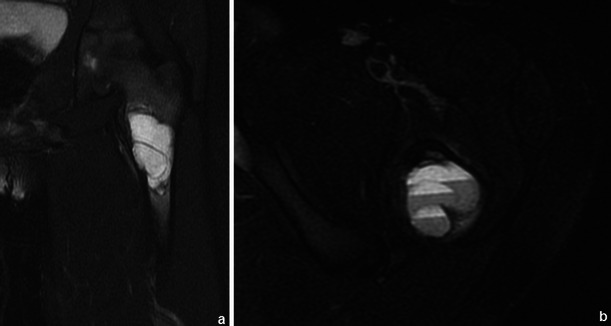
ABC of the left femur: coronal (**a**) and axial (**b**) T2FS WI: Typical fluid-fluid levels are seen, with no intervening soft tissue or thick septations. The cortical area is expanded but not disrupted

**Fig. 15:**
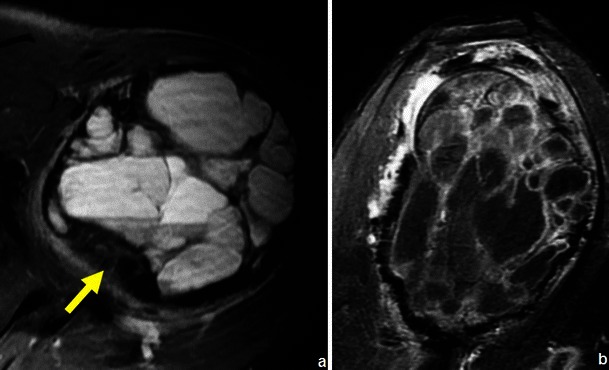
Axial T2WI (**a**) and sagittal post-contrast T1WI (**b**) show typical features of telangiectatic osteosarcoma: a very large large lesion, with a solid component in the posterior dependent part (arrow in **a**) and extension beyond the disrupted cortical region (**b**)

FFLs result from separation of two fluids of differing densities within aneurysmally dilated cavities and generally are most conspicuous on T2WI. They are most common in aneurysmal bone cysts (ABCs) (37– 87 %), but are also frequent in osteosarcomas, giant cell tumours and chondroblastomas [20]. They occur less frequently in lesions such as fibrous dysplasia, osteoblastomas, simple bone cysts and brown tumours [20, 21]. FFL cannot be considered diagnostic of any particular type of tumour, and the diagnosis should be made on the basis of other radiological and clinical findings [21]. The most important differential diagnosis of bone lesions with FFLs is ABC (Fig. 14) vs. telangiectatic osteosarcoma (Fig. 15), because while the former is the most common cause of FFLs, the latter is a high-grade malignant lesion (see Table 3 for MRI aspects useful in the differential diagnosis).

**Table 3:** MRI features that favour the diagnosis of telangiectatic osteosarcoma as opposed to ABC

Solid tissue components surrounding or associated with the cystic/haemorrhagic spaces (better depicted after gadolinium-based contrast medium injection) [24]
Focal solid protrusions through the cortical area or cortical destruction with an associated soft tissue mass [4]

(4) Primary lesions with low signal in T2WI

**Fig. 16:**
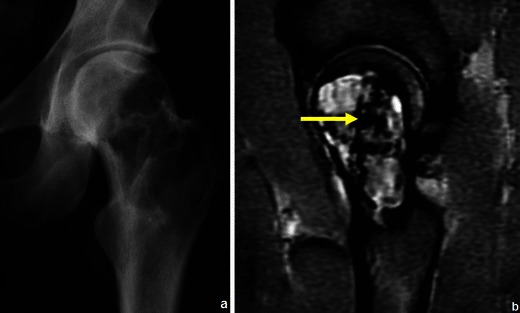
Giant cell tumour of the proximal femur in a 36-year-old patient: the coronal T2WI (**b**) shows a predominantly hyperintense lesion containing areas of low signal intensity; corresponding radiograph in (**a**)

**Fig. 17:**
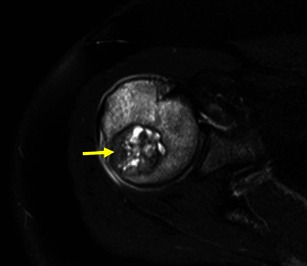
Axial T2FSWI of a chondroblastoma with partial hypointense content (arrow). There is extensive oedema in the adjacent bone, which is typical of these lesions

**Fig. 18:**
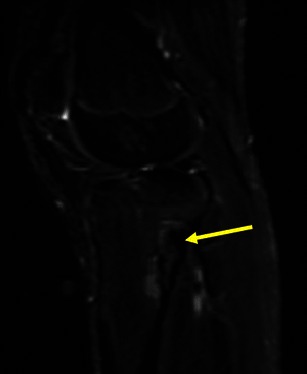
Sagittal STIR image of the knee shows a partial sclerotic component (low signal intensity) within a nonossifying fibroma

Most bone tumours and tumour-like lesions have a significant chondroid/cartilaginous or liquid component, which is associated with high signal intensity on T2WI. There are however primary lesions that can show partial or entire low signal intensity in T2WI because of an immature chondroid matrix, haemosiderin and calcifications [23]. This feature can be used for making the specific diagnosis (Table 4; Figs. 16, 17and 18).

**Table 4:** Primary bone tumours and tumour-like lesions with low signal T2WI

Giant cell tumour	Predominantly hyperintense in T2WI but, in 63 % of the cases, will show low signal areas that occupy ≥20 % of the lesion size [19] (Fig. 16)
Chondroblastoma	86 % lesions have T2 hypointense areas, entirely or partly [23] (Fig. 17)
Natural involution of some lesions	Nonossifying fibroma (Fig. 18)

(5) Evaluation of patients with haematopoietic malignancies and premalignant gammopathies

For patients with Hodgkin’s lymphoma and high-grade non-Hodgkin’s lymphoma, FDG-PET has been established as the imaging modality of choice for staging and monitoring treatment. MRI, however, should be considered in cases with high risk of bone marrow involvement and equivocal findings on PET or extracompartmental tumour growth as well as in patients with potential treatment complications [24].

MRI is a noninvasive technique that can complement bone marrow aspirations (the iliac crest biopsy may be false negative when bone marrow infiltration is focal rather than diffuse) [25].

Radiologic skeletal surveys are still part of the recommended baseline evaluation in multiple myeloma (MM), despite their limitations (they only depict lytic lesions after the loss of over 30 % of the bone mineral density, poorly show anatomic areas such as the ribs, pelvis and spine and are not accurate for the evaluation of diffuse medullary extension) [26]. In several studies, MRI showed a higher sensitivity than skeletal surveys for the detection of focal bone marrow lesions [27, 28]. MRI’s increased diagnostic accuracy led to revising the traditional Durie-Salmon staging system (which only included radiographs in the imaging approach). The newer Durie-Salmon PLUS staging system includes MRI in the evaluation of the spine (summarised in Table 5). Staging and treatment can potentially change in 15–25 % of patients [29].

**Table 5:** New imaging techniques in the Durie-Salmon PLUS staging system [22]

Durie-Salmon PLUS staging system	
Classification	New imaging (MRI and/or FDG PET)
MGUS	All negative (normal marrow)
Stage IA (Smouldering or indolent)	Can have single plasmacytoma and/or Limited disease (definition evolving)
Multiple myeloma	
IB	<5 focal lesions; mild diffuse disease
IIA/B	5-20 focal lesions; moderate diffuse disease
IIIA/B	>20 focal lesions; severe diffuse disease

MGUS: Monoclonal gammopathy of undetermined significance

A: serum creatinine <2.0 mg/dl; no extramedullary disease (EMD)

B: serum creatinine >2.0 mg/dl; extramedullary disease (EMD)

Mild difuse disease: micronodular pattern; moderate disease: diffuse low signal intensity on T1WI, but contrast between the bone marrow and disk remains; severe disease: contrast between bone marrow and disk is lost or inverted (bone marrow shows a signal intensity equal or inferior to that of the disk on T1WI)

The possibility of extending the spinal MRI evaluation to a whole-body MRI (WBMRI) evaluation of the skeleton in MM is under active investigation because a significant percentage of lesions occur outside the axial skeleton [30].

**Fig. 19:**
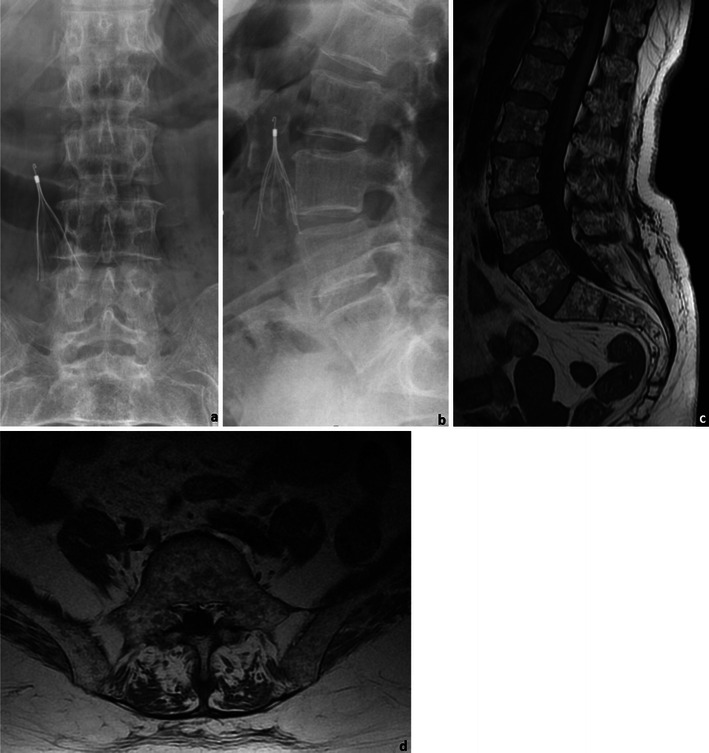
A 76-year-old female patient with MM: Frontal (**a**) and lateral (**b**) radiographs of the lumbar spine and sagittal (**c**) and axial (**d**) T1WI of the lumbosacral spine. The radiographic study shows diffuse osteopaenia of the lumbar vertebrae, which could only be related to primary osteoporosis given the patients’ gender and age. The MRI study however showed innumerous focal lesions, consistent with the diagnosis of MM. Note: The small cone-shaped device present in the radiographs is an inferior vena cava filter

MRI is now widely used in MM not only for assessing the bulk of disease in patients with osteopaenia or uncertain staging (Fig. 19), but also to document the extent of bone marrow infiltration and cord or root compression in patients with pain syndromes [31]. It may also be used to select appropriate biopsy sites [25].

Monoclonal gammopathy of undetermined significance (MGUS) and smouldering multiple myeloma (SMM) are asymptomatic, pre-malignant disorders that can progress to MM. The Durie-Salmon PLUS staging system includes features that allow a clear distinction between MGUS and MM. The MRI findings in SMM are still currently under investigation [8]. In patients with asymptomatic or smoldering myeloma, MRI findings have shown a correlation with the likelihood of transition to MM [29].

Patients with MGUS and SMM require indefinite follow-up given their lifelong risk of progression to MM or related malignancy [32]. A skeletal survey should be repeated at least once every year for SMM [32], but MRI may be better suited for the imaging follow-up.

Despite the improvements that MRI brought to the imaging evaluation of haematopoietic malignancies, there are still limitations regarding sensitivity and specificity. An infiltration with less than 20 % neoplastic cells cannot be distinguished from normal marrow with standard MR pulse sequences [24]. Several authors reported a normal bone marrow MR signal in patients with leukaemia, in patients with early stages of bone marrow invasion by lymphoproliferative diseases and even in up to one-quarter of patients with stage III MM [24, 33]. MRI specificity of signal alterations of bone marrow is low, which is why the findings need to be correlated with clinical and laboratory findings.

Coronal T1 and STIR images of the entire skeleton (WBMRI) can be acquired quickly and provide good morphological evaluation of the extent of the disease. Functional imaging with contrast-enhanced dynamic imaging can provide information regarding disease activity [34]. The future role of these techniques in oncohaematological diseases still needs to be defined, but WBMRI will probably become the bone marrow imaging method of choice.

## Staging malignant bone tumours using MRI

The American Joint Committee on Cancer (AJCC) staging system (Table 6) is currently the most used for malignant bone tumours. It separates stages I and II according to the tumour size (whether  ≤  or  > 8 cm) and defines stage III for cases with “skip metastasis” (discontinuous tumours in the primary bone site). Stage IV is subdivided according to the presence or absence of metastases in locations other than the lung.

**Table 6:** AJCC staging system (adapted from the AJCC Cancer Staging Forms, 7th Edition)

*American Joint Committee on Cancer(AJCC)* staging system				
Stage	Primary tumour (T)	Regional lymph nodes (N)	Distant metastasis (M)	Histologic grade (G)
Stage IA	T1	N0	M0	G1, 2 (low grade)
Stage IB	T2 T3	N0 N0	M0 N0	G1, 2 (low grade)
Stage IIA	T1	N0	M0	G3, 4 (high grade)
Stage IIB	T2	N0	M0	G3, 4 (high grade)
Stage III	T3	N0	M0	G3, 4 (high grade)
Stage IVA	Any T	N0	M1a	Any G
Stage IVB	Any T Any T	N1 Any N	Any M M1b	Any G Any G

### Local staging and preoperative assessment

Both CT and MRI can provide preoperative planning and staging, but existing data suggest that MRI should be the preferred technique [1, 35].

T1WI and CEMRI can accurately depict the extension of the primary lesion. Its exact location should be clearly stated in the report (e.g. measuring the distance of the lesion to an anatomic reference will help planning surgical procedures).

The distinction between tumour and oedema can be difficult, but is essential for local staging and guiding biopsy. Tumoral tissue has a more heterogeneous signal than that of associated oedema. Contrast-enhanced dynamic imaging can also be used (the oedema will show a slope 20 % or less than the tumour itself) [17].

**Fig. 20:**
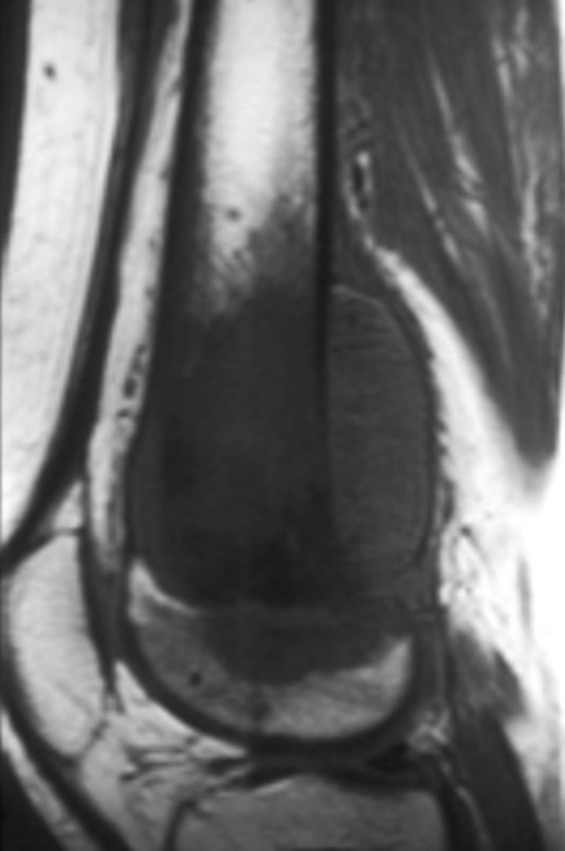
T1W sagittal image of an osteosarcoma of the distal femur showing extension beyond the physeal plate

Osteosarcoma and Ewing’s tumour are more common in paediatric ages. Though the physeal plate was traditionally thought to be a significant barrier for tumoral spread, it is now known that 75-88 % of osteosarcomas show extension to the epiphysis in children [19]. Physeal and epiphyseal involvement (Fig. 20) need to be assessed in order to determine whether joint-sparing surgery can be performed. This should be done using T1WI and T2FS WI/STIR. Abnormal physeal signal intensity, especially when contiguous with and isointense to the primary tumour, is the most important feature [4].

**Fig. 21:**
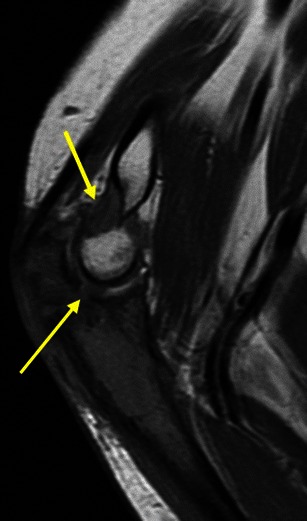
T1W coronal image of an osteosarcoma of the ulna showing extension into the elbow joint with an associated effusion (arrows)

**Fig. 22:**
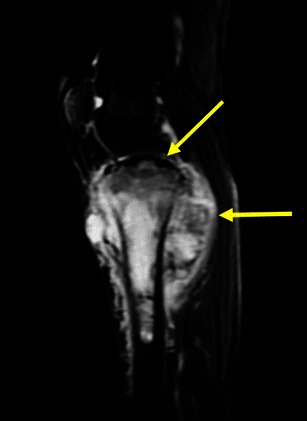
T2FS sagittal image of an osteosarcoma of the proximal tibia: there is muscular invasion (lower arrow) and intra-articular extension (upper arrow)

Direct visualisation of a tumour extending into the articular cavity through a destroyed cortical region is the most obvious sign of joint involvement (Figs. 21 and 22). Joint effusion alone is not sufficient for its diagnosis but its absence can help exclude it with a high degree of certainty [4].

Muscular invasion should preferentially be assessed in the axial plane using T1WI and T2FS/PD FS WI [4].

**Fig. 23:**
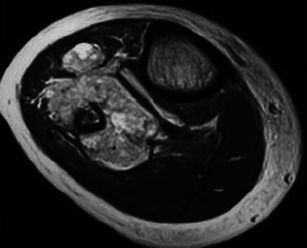
Gadolinium-based contrast medium-enhanced T1W axial image: osteosarcoma of the right tibia invading the adjacent soft tissues (including the vasculature)

MRI is superior to CT and conventional angiography in the evaluation of neurovascular involvement [4]. The best predictors are loss of the perivascular/perineural fat and encasement (especially with associated stenosis). They should be assessed in the axial plane using T2FS/PD FSWI and CEMRI (Fig. 23).

### Use of MRI in evaluating distant extension

**Fig. 24:**
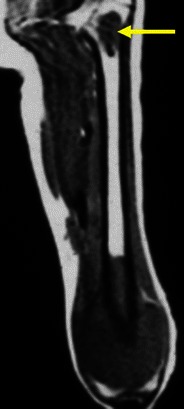
Coronal T1 image of an osteosarcoma of the distal femur with an associated proximal skip lesion (arrow)

Skip metastases (Fig. 24) are considered rare (less than 5 % of osteosarcomas [19]); however, their presence automatically determines an AJCC stage ≥III and is considered a poor prognostic sign [36].

Transarticular skips should be considered stage IV disease (distant metastasis) because aggressive chemotheraphy (used for typical skip metastases, stage III) does not improve diseased-free survival in these patients [37].

MRI has no role in assessing lymph node or lung involvement. It is currently under active investigation as an alternative to bone scan or FDG-PET in the assessment of bony metastasis.

## Assessing response to neoadjuvant therapy with MRI

Chemotherapy has an important role in the treatment of MM, osteosarcoma, Ewing sarcoma’s and lymphoma.

Most bone tumours are resistant to radiotherapy [28]: its role is limited to local treatment of Ewing’s sarcoma [38], some cases of conventional osteosarcoma (when marginal resection is required for functionality [19]) and chordoma [16].

Chondrosarcomas and malignant fibrous histiocytoma are usually resistant to both chemotherapy and radiotherapy [16, 38].

Expected post-radiation MRI changes in successful cases are a decrease in tumour size and an increase in its T2 signal intensity (due to fatty transformation of the bone marrow).

**Fig. 25:**
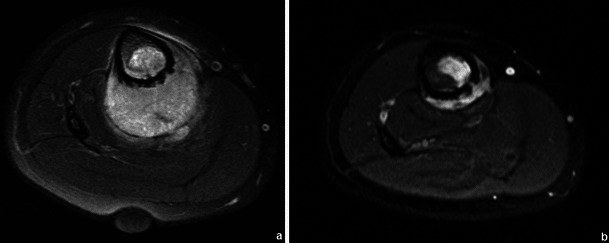
Ewing’s sarcoma before (**a**) and after (**b**) chemotherapy: a decrease in the size of the soft-tissue component and presence of a circumferential hypointense rim are features suggestive of good response

Response to preoperative chemotherapy in bone sarcomas can be classified as good (≥90 % tumour necrosis) or poor (<90 % tumour necrosis) [39]. Decreasing extent of marrow invasion, reduction of tumour volume and a decrease in the amount of associated oedema are favourable conventional MRI indicators (Fig. 25). Quantitative dynamic MRI can estimate the amount of necrosis in bone tumours (viable tumour enhances faster than nonviable tumour and post-treatment changes [40]). If confirmed to be of clinically predictive value, the post-processing software needed for performing this technique would likely become more available [4]. Favourable indicators in dynamic CEMRI are reduction in the slope of the time intensity curve, of the maximum enhancement and in the washout rate. These can change the type of signal enhancement time curve [4].

Necrotic tumour does not restrict the movement of water molecules (this translates into low signal intensity in high b values and a high ADC in DWI) as opposed to viable tumour.

## MRI applied in follow-up

Evaluation for osseous metastatic disease should only be done in symptomatic patients [10]. Coronal whole-body and sagittal spine MRI using T1WI and STIR were shown to be superior to FDG-PET/CT for this purpose [41]. The combined use of radiographs and MRI is strongly recommended for the surveillance for local recurrences [10]. Baseline imaging should be obtained within 3-6 months of definite resection using MRI [19]. Recommended imaging follow-up intervals vary according to whether the tumour is low or high risk [10].

## Detection of recurrences and distinction from posttherapeutic changes

Rooser et al. [42] demonstrated marginal excision, tumour necrosis and extracompartmental extension to be the most important risk factors for local recurrences.

Recurrences should be suspected when residual bone changes occur, such as marrow replacement, cortical disruption and osseous destruction. When performing an MRI follow-up study, lesions should first be evaluated according to their T2 signal intensity: if it shows low signal intensity on T2, it generally does not represent recurrent tumour (sensitivity 96 %) [10]. If it shows high signal on T2 and surgery was the only treatment performed, the likelihood of recurrence is high. When radiotherapy was also carried out, the high signal on T2 is nonspecific for distinguishing recurrence or radiation-induced inflammation [43].

**Fig. 26:**
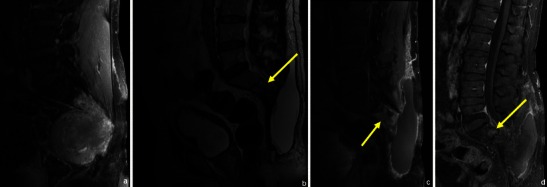
MRI applied in the diagnosis and follow-up of a patient with chordoma: this patient had a voluminous lesion destroying the distal part of the sacrum (**a**). He had radiotherapy and surgery and was re-evaluated 4 months later: MRI showed a posterior subcutaneous fluid collection and a localised area with high T2WI signal (arrow in **b**) and contrast enhancement (arrow in **c**) in the inferior spine, which could be due to either residual tumoral tissue or radiotherapy. Posterior follow-up however showed a localised round area of persistant enhancement, which proved to be local recurrent/residual tumour (arrow in **d**)

**Fig. 27:**
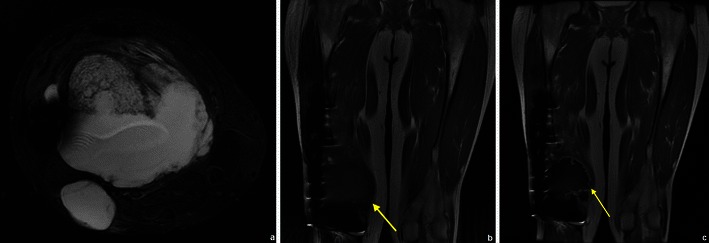
This patient had resection of the distal femur due to a chondrosarcoma. MRI showed a suspicious area of high signal near the distal prosthesis (**a** and arrow in **b**), which showed peripheral enhancing lobulations (arrow in **c**): a local recurrence of chondrosarcoma

Postoperative fluid collections (haematoma and seroma) can be distinguished from residual tumour or inflammation by means of CEMRI (Figs. 26 and 27): the former will show only thin linear peripheral enhancement.

**Fig. 28:**
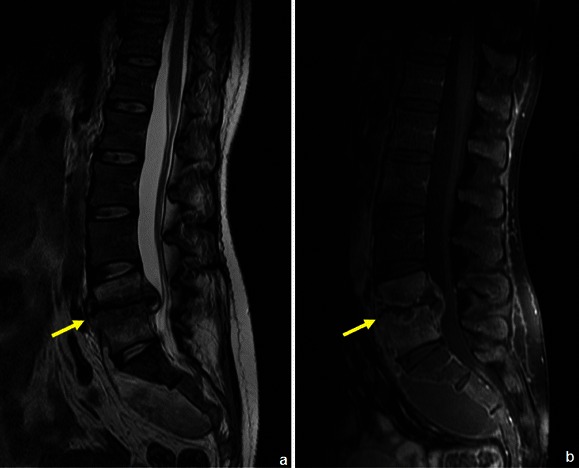
L4-L5 spondylodiscitis: Sagittal T2 (**a**) and sagittal CEMRI (**b**) show intervertebral disk and body endplate destruction (arrows in **a** and **b**), oedema of the paraspinal musculature and a large liquid collection inferiorly. The infection extends posteriorly into the spinal canal. Primary bone lesions usually do not contiguously involve adjacent vertebrae

**Fig. 29:**
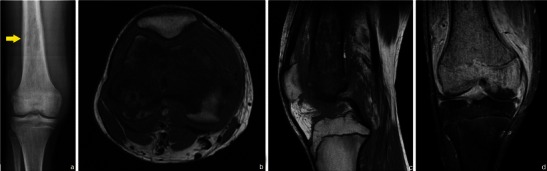
Acute osteomyelitis radiographic findings (**a**) can be aggressive and similar to those of Ewing’s sarcoma (permeating lesion with periosteal reaction, arrow in **a**). MRI however shows diffuse soft-tissue swelling, with no well-defined soft tissue mass (**b** axial T1WI; **c** sagittal T1WI; **d** contrast-enhanced T1WI)

**Fig. 30:**
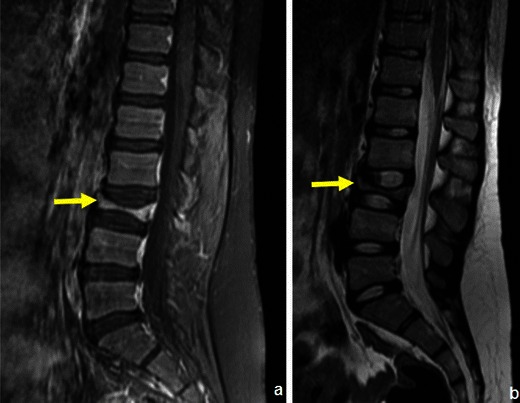
Sagittal T1- and T2WI of a typical eosinophilic granuloma of the spine (vertebra plana): preservation of terminal end plates, disks and posterior elements

**Fig. 31:**
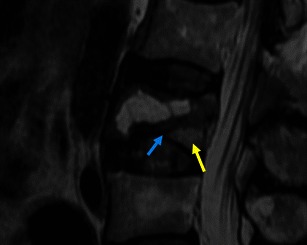
Sagittal T2WI: typical benign insufficiency vertebral fracture with a visible fracture line (blue arrow), areas of normal marrow (yellow arrow), and no focal lesion or associated mass

**Fig. 32:**
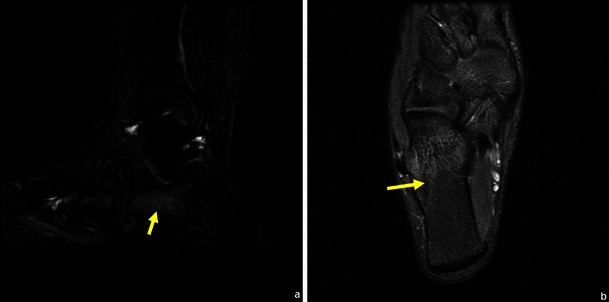
Stress fracture of the calcaneus: sagittal and axial STIR shows an area of localised marrow oedema (arrow in **a**) and the hypointense fracture line (arrow in **b**) extending from the cortical to the medullary area

**Fig. 33:**
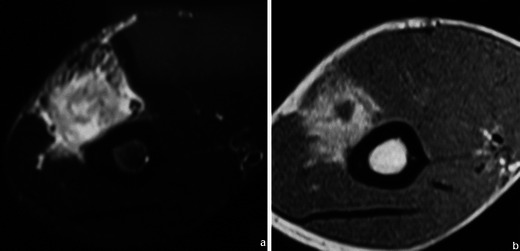
Acute/intermediate justacortical myositis ossificans of the right thigh: axial STIR (**a**) shows a localised high signal intensity area with associated oedema. Contrast-enhanced T1WI (**b**) demonstrates enhancement, which is typical of active lesions. This lesion does not disrupt the cortical area

**Fig. 34:**
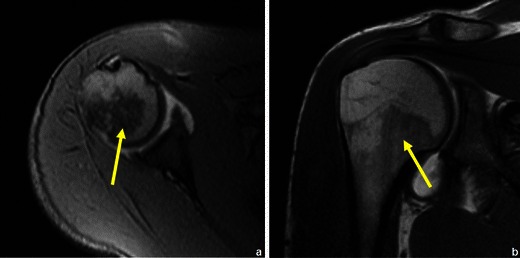
Normal haematopoietic marrow in the proximal humerus (arrows in **a** and **b**). In children, the normal bone marrow is highly cellular (low signal on T1WI and high signal T2 FS WI). With increasing age, a gradual conversion from this highly cellular marrow to fatty marrow occurs (with an increase in the bone marrow signal on T1WI and a decline on T2 FS WI). In long bones, this conversion first involves the epiphyses, then the diaphyses and finally the metaphyses [24]

**Fig. 35:**
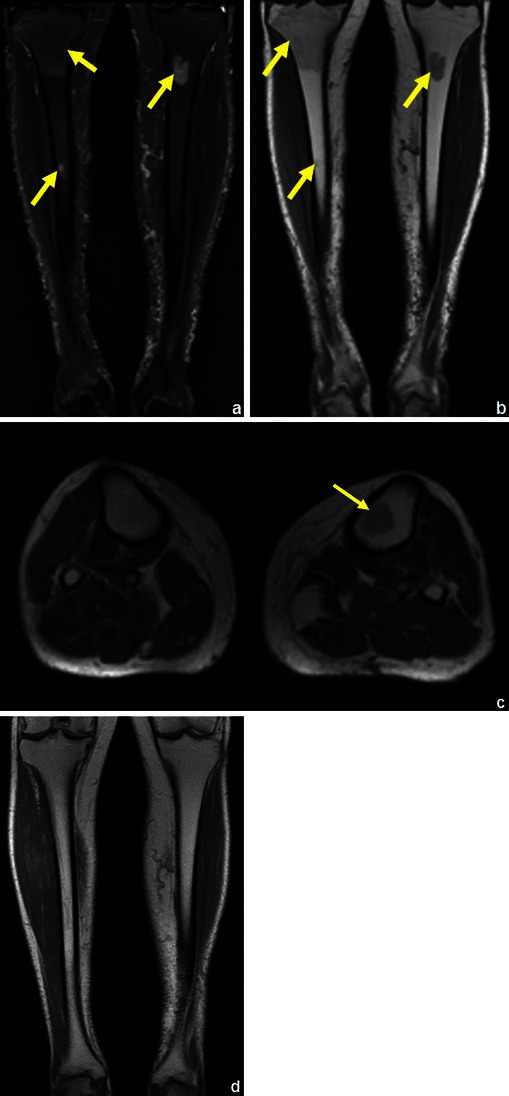
A patient with a history of lung cancer: coronal STIR (**a**) showed several suspicious areas on both tibial bones (arrows in **a**), which could be misinterpreted as distant recurrence if not noticed to show higher T1 (arrows in **b** and **c**) signal than adjacent muscle. Posterior follow-up (**d**) showed complete resolution of a these areas. This reconversion occurs in a reverse fashion compared to the conversion from haematopoietic marrow to fatty marrow (i.e. the reconversion progresses from the central skeleton to the periphery) [24]

**Fig. 36:**
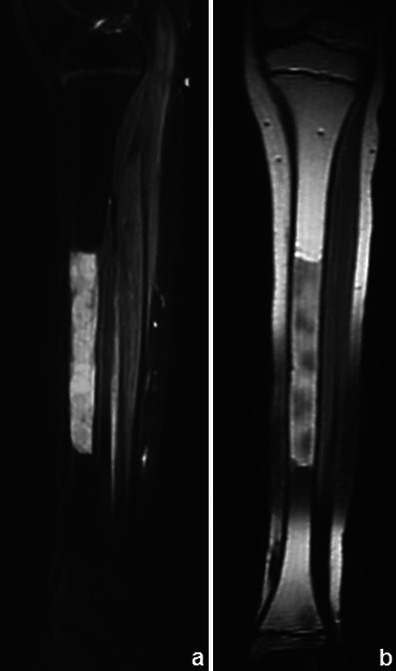
Medullary rebound in a young female patient with a history of recent chemotherapy for treatment of Ewing’s sarcoma: coronal STIR (**a**) showed an area of increased intensity in the tibial diaphysis that displayed higher signal intensity than the muscle on T1WI (**b**)

Chronic, post-therapeutic changes usually lack high signal intensity on T2WI. The presence of vascularised granulation tissue, neovascularity in necrotic areas or reactive hyperaemia can cause gadolinium-based contrast medium enhancement. However, because of its greater vascularisation, tumour tissue normally enhances more.

Dynamic CEMRI may be beneficial by demonstrating early enhancement in tumour tissue that is not seen in post-therapeutic changes or inflammation.

## Important diagnostic differentials and mistakes that should be avoided

There are many nontumoral (e.g. infectious and traumatic) conditions that can look like primary bone tumours (“mimickers”). There is also a group of situations that can lead to errors in staging or incorrect diagnosis of recurrence (and thus can be regarded as pitfalls). Although their detailed discussion is beyond the scope of this work, some of them should be mentioned and are briefly discussed in Tables 7and 8 and (Figs. 28, 29, 30, 31, 32, 33, 34, 35, and36) [15, 19, 24, 44–46].

**Table 7:** Mimickers

Condition	Explanation	Distinguishing features/recommendations
Osteomyelitis (Figs. 28 and 29)	Aggressive aspect	Usually involves the metaphysis in children Small/absent soft tissue component Penumbra sign* Fistulous tracts - clinical features**
Eosinophilic granuloma (EG)	Aggressive aspect	Can be very difficult or even impossible to distinguish from malignant lesions in young patients EG of the spine shows preservation of terminal end plates, disks and posterior elements (Fig. 30)
Stress lesions (Figs. 31 and 32)	-Elderly patients with insufficiency fractures , fatigue fractures	Hypointense line extending from the cortex into the medullary area on T1- and T2WI Surrounding oedema [15] , Absence of focal lesion or soft tissue mass [15]
Bone infarcts and osteonecrosis	Early osteonecrosis may result in a poorly defined region of lucency simulating a tumour in radiographs , Calcifications can simulate those of chondroid lesions	Usually manifests as a well-defined linear serpentine rim of low signal intensity on T1WI. On T2WI the rim may have low signal intensity, high signal intensity or both (“double line” sign) Chondroid lesions show peripheral lobulations with T2 hyperintensity
Myositis ossificans (Fig. 33)	Can have a disorganised amorphous bone formation similar to osteosarcoma [19, 44]	Usually separated from the cortex Evolves to mature ossification
Haematopoietic marrow (Fig. 34)	Axial skeleton, thoracic grid, pelvis and extremities of long bones can maintain areas of haematopoietic marrow even after skeletal maturity [15] (this can simulate marrow infiltration in T1WI and cause high signal on STIR)	Signal intensity > than that of muscle on T1WI Presence of microscopic fat (>50 % drop in signal in opposed-phase) [15]
Aggressive osteoporosis	Sudden immobilisation can cause bone demineralisation, with oedema mimicking diffuse tumoral infiltration	Preferential locations: subchondral bone, tendon and ligamentar insertions

*The “penumbra sign” on MRI is a rim with higher signal intensity than that of the main abscess on T1WI. It is helpful in distinguishing between subacute osteomyelitis from other osseous lesions [45]

**Rapid onset of fever, localised pain and oedema; 50 % of cases show positive blood cultures

**Table 8:** Pitfalls

Pitfall	Explanation	Distinguishing features/ recommendations
Very infiltrative lesions (e.g. MM)	Can preserve bone marrow fat [15] (normal signal on T1WI and signal drop in out of phase)	Can be undetectable
Anaemia, rebound following chemotherapy or treatment with colony-stimulating factor (Figs. 35 and 36)	Marked haematopoiesis increases the amount of red marrow, resembling recurrent tumoral disease	Usually bilateral and symmetric -Signal intensity > than that of muscle on T1WI USPIO-enhanced MRI can differentiate these from tumour deposits (RES cells are present in the reconverted marrow but not present/substantially reduced in tumour deposits [24]
Post-chemotherapy osteosarcoma “size increase”	Sometimes even in “good responders” the primary lesion does not diminish in size or appears to enlarge	Therapy has low impact on the mineralised matrix of osteogenic sarcoma Matrix maturation/ossification (presence of fatty marrow)
Benign GCT may show elevated choline levels on proton MR spectroscopy [46]	May be related to the degree of their local aggressiveness	Use radiographs and conventional MRI for diagnosis

USPIOs: ultra-small superparamagnetic iron oxide particles

RES: reticuloendothelial system

## Limitations

General MRI contraindications also apply to the evaluation of bone tumours and tumour-like conditions (e.g. patient size, clinical status, cardiac pacemaker).

Low-risk Gd-based contrast agents [47] should only be used if they can provide essential diagnostic information in patients with a glomerular filtration rate <30 ml/min and in pregnant women [47].

Tumour recurrence may be hard to detect when orthopaedic implants are in close proximity to tumour sites because of susceptibility artefacts.

MRI is limited in evaluating calcifications. CT plays a further role in the characterisation of sclerotic or mixed (lytic/sclerotic) lesions and is superior to MRI in the evaluation of osteoid osteoma [10, 35].

## Conclusion

A good knowledge of the characteristic MRI findings of benign and malignant osseous conditions and their role in staging, therapeutic planning and follow-up in the setting of malignancy is essential for optimal patient care.
